# Visual experience shapes the Bouba-Kiki effect and the size-weight illusion upon sight restoration from congenital blindness

**DOI:** 10.1038/s41598-023-38486-y

**Published:** 2023-07-15

**Authors:** Sophia Piller, Irene Senna, Marc O. Ernst

**Affiliations:** 1 Applied Cognitive Psychology, Faculty for Computer Science, Engineering, and Psychology, Ulm University, Ulm, Germany; 2 Transfer Center for Neuroscience and Education (ZNL), Ulm University, Ulm, Germany; 3 Department of Psychology, Liverpool Hope University, Liverpool, UK

**Keywords:** Human behaviour, Neuroscience

## Abstract

The Bouba-Kiki effect is the systematic mapping between round/spiky shapes and speech sounds (“Bouba”/“Kiki”). In the size-weight illusion, participants judge the smaller of two equally-weighted objects as being heavier. Here we investigated the contribution of visual experience to the development of these phenomena. We compared three groups: early blind individuals (no visual experience), individuals treated for congenital cataracts years after birth (late visual experience), and typically sighted controls (visual experience from birth). We found that, in cataract-treated participants (tested visually/visuo-haptically), both phenomena are absent shortly after sight onset, just like in blind individuals (tested haptically). However, they emerge within months following surgery, becoming statistically indistinguishable from the sighted controls. This suggests a pivotal role of visual experience and refutes the existence of an early sensitive period: A short period of experience, even when gained only years after birth, is sufficient for participants to visually pick-up regularities in the environment, contributing to the development of these phenomena.

## Introduction

Experience drives how we perceive the world^1–3^. Two phenomena that have been described as resulting from visual experience with the world are the “Bouba-Kiki” effect and the size-weight illusion (SWI).

In the SWI, participants judge the smaller of two equally-weighted objects to be heavier^4,5^. In the classic SWI, the size of the object is estimated visuo-haptically (i.e. holding and looking at it simultaneously), but the illusion has also been reported in purely visual or haptic conditions^6–8^. The SWI is robust, unaffected by the explicit knowledge that both objects have equal weight^9^. Although different mechanisms underlying the illusion have been suggested^9–11^ and its origin remains unclear, it has been shown that it is not purely sensory but inherits a cognitive component^9,12^. An essential precondition for the illusion to occur is the expectation that one of the objects is heavier than the other^12^. This expectation is shaped by experience with the natural environment in which, typically, the size of an object covaries with its weight^13^. However, if an individual is exposed to an inverted pattern for a prolonged time (i.e. where larger objects are lighter than smaller ones), the SWI can disappear and finally even reverse^2^. Apparently, the experience with the environment leading to the development of the illusion does not necessarily have to be visual, since the haptic SWI has been found in congenitally blind individuals^8,14^. Developmental studies could shed light on the role of experience in the development of the SWI. However, surprisingly little research has been conducted in typically developing children, and results of the few existing studies (most of them dating back more than 50 years) are contradictory^15–20^. More recent reports show that the illusion is already present in pre-schoolers^18^ and strengthens with age^15^, but it is unclear when it emerges or reaches adult-like levels. A recent study suggests that early visual input might not be essential for the illusion to develop, as even individuals treated for congenital cataracts several years after birth are susceptible to the illusion^14^.

While the correlation between object size and weight is more evident in everyday life, people also make other associations between inputs from different sensory modalities that seem more arbitrary: e.g. matching pitch or color with object shape, size, or weight (e.g.^21–23^). One example of such crossmodal correspondences is the sound-shape correspondence, as in the “Bouba-Kiki” effect, where participants consistently match round or spiky shapes with the nonsense words “Bouba” and “Kiki”, respectively^24,25^. The effect is persistent across languages and cultures^26–28^ with only very few exceptions^29,30^, and it has been described even in toddlers^31^ and infants as young as 4 months^32^. This seeming universality of the effect has led to the hypothesis that it might be innate^33^, based on connections between primary sensory areas^25,33^. However, some recent reports surprisingly failed to show the Bouba-Kiki effect in early blind subjects^33,34^, and in individuals treated for congenital cataracts after a prolonged period of visual deprivation^33^, which strongly contradicts this account. Another possibility is that it is learned based on statistical regularities in the environment, in which certain phenomena often co-occur^3,23,34–36^. Vision allows individuals to perceive these regularities that might not be readily available to blind individuals. If this is the case, the question arises as to whether visual experience in the first years of life is essential for developing phenomena like the Bouba-Kiki effect and the SWI, or whether visual experience gained only later in life, after years of visual deprivation, can still drive their development. Here, we investigated the role of early and late visual experience for the development of these two phenomena in late cataract-treated individuals and in the typically sighted population.

## Results

We investigated the Bouba-Kiki effect in the visual modality in cataract-treated participants and in sighted controls, and in the haptic modality in blind participants and sighted controls (see “Methods”). For each condition (visual, haptic) we used logistic regression models to compare the proportion of the consensual association among groups, and to test whether the prevalence of the consensual association in each group differed from chance levels (see “Methods”).

**Figure 1:**
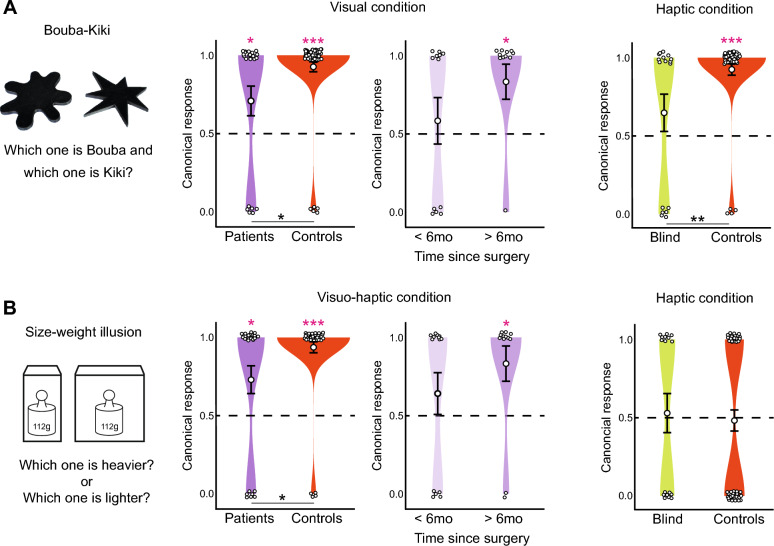
Bouba-Kiki effect and Size-weight illusion (SWI). We measured the Bouba-Kiki effect (**A**) and the SWI (**B**) in cataract-treated patients, blind individuals, and sighted controls. (**A**) Left: To test for the Bouba-Kiki effect, one 3D-printed shape with a rounded outline and one spiky shape were presented to participants either visually or haptically. Participants had to decide which shape was called “Bouba” and which one was called “Kiki”. Middle: When presented visually, most cataract-treated patients (purple) and controls (red) reported the consensual association, i.e. labeled the round shape as “Bouba” and the spiky one as “Kiki”. Controls showed the effect more often than patients. No Bouba-Kiki effect was present in patients tested less than six months after cataract removal (light purple, performance not different from chance). Six months past surgery, the Bouba-Kiki effect was indistinguishable from that of controls (darker purple). Right: When presented haptically, most controls (red) experienced the Bouba-Kiki effect, while blind individuals (green) did not differ from chance level. (**B**) Left: Participants were presented with two objects of equal weight but different size and had to decide which one felt heavier or lighter. The SWI was present if they indicated the small object to feel heavier or the large one to feel lighter. Middle: When presented visuo-haptically, most cataract-treated patients (purple) and sighted controls (red) were susceptible to the SWI. Overall, controls showed the effect more often than patients. We did not find the SWI in patients tested less than six months after cataract removal (light purple, performance not different from chance). Six months after surgery, the SWI was indistinguishable from that of controls (purple). Right: When objects were presented purely haptically, neither controls (red) nor blind individuals (green) did experience the illusion. Stars indicate significance levels (*p < 0.05, **p < 0.01, ***p < 0.001). Dashed lines indicate chance performance. Small white circles indicate individual responses (1: consensuall association; 0: non-consensuall association). Large white circles indicate mean group performance. Error bars indicate the standard error of the mean.

We found that the Bouba-Kiki effect was present in both cataract-treated patients and sighted controls in the visual condition. Both groups made the consensual choice more often as would be expected by chance (patients: mean = 0.74, z = 2.27, *p* = 0.02; controls: mean = 0.93, z = 6.54, *p* < 0.001, all mean values indicate the proportion of the consensual association, Fig. 1A). Still, the groups significantly differed from each other, with controls showing the effect more often than patients (z = 2.32, *p* = 0.02, Fig. 1A). When taking the time of visual experience after sight onset into account, i.e. time that had passed since the surgery, we found that patients with less than six months of visual experience did not differ from chance level (mean = 0.58, z = 0.58, *p* = 0.57), while the ones with more experience did (mean = 0.91, z = 2.20, *p* = 0.03, Fig. 1A). Patients that were tested more than six months after surgery no longer differed from controls (z = 0.24, *p* = 0.81). The small cohort and the binary nature of the data did not allow for a more refined analysis of time since surgery on an individual participant basis. However, in previous studies it was found that several skills mostly develop within the first six months after cataract surgery^37,38^. Thus, it seems reasonable to use the same timeframe to divide the participants into two groups in the present study.

In the haptic condition, blind individuals did not show the Bouba-Kiki effect (mean = 0.65, z = 1.19, *p* = 0.23, not different from chance), while controls did (mean = 0.93, z = 4.82, *p* < 0.001). Thus, the two groups differed, with controls showing the effect significantly more often (z = 2.61, *p* = 0.01, Fig. 1A). There was no significant effect of age in the sighted control group in either condition (visual condition: z = 1.4, *p* = 0.17; haptic condition: z = 0.12, *p* = 0.91): consistent with previous studies^31–33^, already the youngest children (4 years old) consistently showed the effect.

We tested the size-weight illusion visual-haptically in cataract-treated and sighted controls and in the haptic modality in blind participants and sighted controls (see “Methods”). We assessed group differences and differences from chance level with logistic regression models. Both cataract-treated patients and controls experience the SWI in the visuo-haptic condition. Both groups reported more often that the smaller object felt heavier than it would be expected by chance (patients: mean = 0.73, z = 2.26, *p* = 0.02, controls: mean = 0.93, z = 4.86, *p* < 0.001, Fig. 1B). Still, controls showed the effect significantly more often than patients (z = 2.24, *p* = 0.03, Fig. 1B). Again, patients tested less than six months after surgery did not experience the illusion (mean = 0.60, z = 0.77, *p* = 0.44, not different from chance level), while the ones tested more than six months after surgery did (mean = 0.91, z = 2.20, *p* = 0.03, Fig. 1B). Patients that were tested more than six months after surgery no longer differed from controls (z = 0.34, *p* = 0.74). We did not find a significant effect of age in the sighted control group in the visuo-haptic condition of the SWI (z = 1.89, *p* = 0.06).

**Figure 2:**
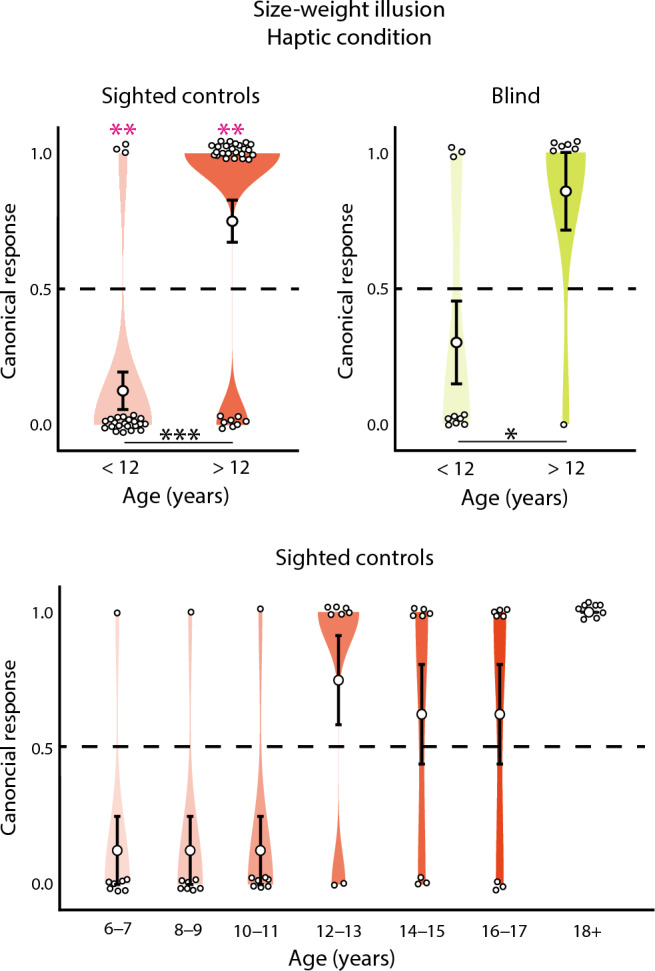
Haptic size-weight illusion (SWI) as a function of age. When tested haptically, sighted control participants under the age of 12 years (light red), consistently judged the larger of two equally weighted but differently sized objects to be the heavier one. Over the age of 12 years, participants (red) chose the small object to feel heavier (i.e. experienced the SWI). Similarly, blind individuals under the age of 12 years (light green) experienced the SWI less often than blind individuals over the age of 12 years (green). In the sighted control group, age as a continuous variable was a significant predictor of the SWI in a logistic regression (different age groups within the group of sighted controls represented by different shades of red). Stars indicate significance levels (*p < 0.05, **p < 0.01, ***p < 0.001). Dashed lines indicate chance performance. Small white circles indicate individual responses (1: consensual response; 0 non-consensual response). Large white circles indicate mean group performance. Error bars indicate the standard error of the mean.

In the haptic condition, on average neither controls (mean = 0.48, z = 0.27, *p* = 0.79), nor blind individuals (mean = 0.53, z = 0.24, *p* = 0.81, Fig. 1B) were susceptible to the illusion. This was surprising, given that the illusion has previously been reported in purely haptic settings in both sighted and blind individuals^8,14^. However, contrary to our study, these studies only included adult participants. Thus, we analyzed whether there were any systematic effects of age in blind or sighted participants. Indeed, we found a significant effect of age in the sighted controls (z = 3.63, *p* < 0.001). As depicted in Fig. 2, participants under the age of 12 years systematically choose in the opposite direction, i.e. reported that the larger object felt heavier (mean = 0.13, z = 3.15, *p* = 0.002). Older participants chose the consensual decision in the majority of cases (mean = 0.75, z = 2.69, *p* = 0.01). The groups of younger and older participants differed significantly (z = 4.11, *p* < 0.001). In blind individuals, the same pattern as in controls emerged: blind participants over the age of 12 years experienced the illusion significantly more often than the ones below twelve years (z = 2.06, *p* = 0.04, Fig. 2).

To rule out the possibility that any differences found were simply due to language, culture or language barriers, the results of the Ethiopian and German sighted controls were compared in each experiment. As expected, the performance of the controls from both countries did not significantly differ (Bouba-Kiki: z = 0.10, p = 0.92; SWI: z = 0.88, *p* = 0.38).

To rule out that the effects found in the cataract treated patients in both experiments were mainly due to improvements in visual acuity in the months and years after visual recovery, we were able to test the visual acuity of a subset of 27 patients twice: once up to six months after surgery (mean: 1 month, range: 0 days to 6 months) and once more than six months after surgery (mean: 1.5 years, range: 10 months to 4 years). Visual acuity (measured as contrast sensitivity function (CSF), see “Methods”) did not differ significantly between the two measurements (test 1 = mean: 3.6 cpd, range: 0.04–13.2 cpd; test2 = mean: 4.3 cpd, range: 0.04–14.6; Wilcoxon signed-rank test, z = 0.78, *p* = 0.44).

## Discussion

We investigated how early and late visual experience influences the Bouba-Kiki effect and the SWI in sighted, blind, and late cataract-treated individuals. To fully appreciate the possible contribution of late visual experience, we investigated the development of both phenomena over time following cataract-removal surgery.

We did not find the Bouba-Kiki effect in the first few months after surgery. In line with previous studies^33,34^, the effect was absent also in our blind participants. It has been suggested that haptic shape perception evokes visual imagery^39^, and visual imagery might be a precondition for the Bouba-Kiki effect to occur also in purely haptic conditions^34^. As visual imagery is obviously not available to congenital blind individuals, this might explain the lack of the effect in this group. Moreover, blind individuals have to rely on haptics to perceive shape, which might be less efficient than vision in extracting the shape of an object: as haptic exploration typically requires following the object contours over time^40^, it is slow and limited in precision^41,42^. Contrary, visual perception is holistic and allows gathering information about object geometry quickly and precisely^41,42^ and thus might facilitate the reception of environmental regularities that are necessary for the Bouba-Kiki effect to emerge. Other cross-modal correspondences such as pitch-size and pitch-weight are indistinguishable between blind and sighted adult individuals^22^, and others only emerge in blind adults but not in sighted ones (pitch-texture, pitch-softness)^22^. This might reflect the decent accessibility of size, weight, and texture information through haptic exploration^43,44^ compared to shape information. Interestingly, it has been shown that both blind and sighted participants tend to verbally report the color red as heavier than yellow, even though the tested blind individuals had no perception of visual memory of colors^45^. However, the association was stronger in sighted individuals^45^. These results highlight both the role of semantic patterns that can be involved in such associations, but also the important role of sensory experience^45^. It has also been suggested that orthographic features, present in written language but not in Braille, might enhance the Bouba-Kiki effect in sighted compared to blind individuals^46^. For example, the letters “B” and “O” have relatively round outlines, compared to more spiky letters like “I” and “K”, which mimic the round and spiky object shapes used to test the Bouba-Kiki effect. This difference is not present in Braille^46^. However, even after surgery, our cataract-treated sample attended blind schools and used only Braille for reading and writing. Nevertheless, after only six months, their Bouba-Kiki effect was no longer distinguishable from that of typically sighted controls. Thus, we assume that the influence of orthographic features is relatively small compared to the abundance of other relevant visual features in the environment.

A previous study found that the Bouba-Kiki effect was absent in cataract-treated individuals^33^ and concluded that there is an early sensitive period in which patterned visual input is essential for the Bouba-Kiki effect to develop. Our findings contradict this earlier conclusion and suggest that visual experience, even when acquired only later in life, is sufficient to pick up the environmental regularities required to develop the effect. Further studies are needed to explain the opposite results we found. However, we suggest that, as we used black solid objects against a white background instead of subtle outlines creating hollow objects as in^33^, our stimuli could have been easier to see and interpret. Moreover, the contours of our “Kiki” shape presented much deeper protrusions and indentations than the shape in^33^, probably resulting more visible and salient for low-vision patients.

In the SWI experiment, we found the visuo-haptic illusion in both cataract-treated patients and controls. In line with our results, one previous study found that late cataract-treated individuals are susceptible to the SWI^14^. The authors concluded that there is no early sensitive period in which patterned visual input is necessary for the illusion to develop. However, as all individuals tested in^14^ underwent cataract surgery more than six months before getting tested, the contribution of the visual experience gained shortly after surgery could not be investigated. While cataract-treated individuals are susceptible to some visual illusions right after sight onset^47^, other abilities have been shown to develop in the months after surgery^37,48^. Thus, we also included patients that were tested shortly after surgery to take the time of visual experience after cataract removal into account. We found that in patients tested less than six months after surgery, i.e. with little visual experience, the illusion was absent, like in the blind group. Patients that had the possibility to gain some visual experience, i.e. those operated more than six months prior to testing, did fall for the SWI, and no longer differedfrom sighted controls. Thus, our results suggest that, while early visual experience is not essential for the illusion to develop, it is the visual experience gained within a few months after restoration that is critical for its development.

Interestingly, on average across ages, the SWI was also absent in the blindfolded sighted controls that we tested purely haptically. Thus, even the possibility to use visual imagery did not enable controls to experience the illusion. Previously, the SWI has been reported in blindfolded controls^6,8^, as well as in early-blind individuals^8,10,14^, when object size was made available haptically or through echolocation. However, all individuals tested in the aforementioned studies were at least 16-years-old, while the age range in the present study started at six years. Surprisingly, little research has been conducted concerning the development of the SWI in typically developing children and results are contradictory. The existing research suggests that the visuo-haptic SWI is present in school-aged children^15–20^ but might develop even earlier at two or three years of age^18,20^, and there is some evidence that the illusion strengthens with age^15^. This is in line with our results, as we also found the illusion from age six, when tested visuo-haptically. A previous study showed that the SWI tested purely haptically was present from age six and further increased with age^19^. This increase is in line with our findings. However, in contrast, we found that in participants younger than 12 years the haptic illusion was not only absent (i.e. performance at chance level), but it was systematically inverted, i.e. children judged the larger object to be heavier than the smaller one, as it has been previously reported for blind children and adolescents^16^. A possible explanation for the inverted SWI arises when considering a Bayesian framework^10,49^. In the Bayesian approach, prior knowledge (retrieved from environmental statistics) is integrated with the present sensory evidence. As the weight of an object typically covaries with its size, one would expect the larger of two equally-weighted objects to feel heavier. As the opposite is the case in the SWI, it has been labeled “anti-Bayesian”^10,49,50^. However, the SWI might not be anti-Bayesian after all, if instead of the weight, the density is considered. As smaller liftable objects usually are denser than larger ones^13^, that would lead to the prediction that the smaller one should feel heavier, as is the case in the SWI^10,11,51^. While an understanding of the concept of weight develops early on in childhood^20^, density is a complicated, higher-order concept that takes years to mature^52–54^. While some implicit notions of density, (i.e. whether an object will sink or float) starts to emerge around 4–5 years of age^52^, it takes until age 5–7 to establish a more profound knowledge (e.g. something is heavy for its size)^54^ and until age 12 to be really able to disentangle the concepts of size, weight, and density^53^. Moreover, the relationship between object density and weight might be easier to grasp visually than haptically, since some object properties (bottle is full or empty, object is solid or has holes, material) may not be readily available to haptics. One could speculate that the lack of vision, as in blindfolded, blind participants, or patients before cataract removal, until a certain age impairs density estimation. Thus, if we assume that children either use a cognitive strategy (i.e. “the larger object should weigh more because it is larger”), or judge the weights within a Bayesian framework, both would lead to the participants consistently judging the larger object to be heavier.

Our findings suggest that vision and visual experience play a major role for both the Bouba-Kiki effect and the SWI, especially in children. Importantly, the human brain can quickly pick-up certain regularities in the environment that are the basis of both phenomena, even after years of visual deprivation. The timeframe of the found improvements is consistent with evidence from cataract-treated patients showing that multisensory integration also appears to develop around six months after surgery^37^. Similarly, dark-reared cats develop multisensory enhancement at the single neuron level in a similar timeframe to our patients^55,56^**.**

Our results refute the presence of an early sensitive period in which patterned visual input is essential for the Bouba-Kiki effect or the SWI to emerge. Instead, we show that visual experience, even when occurring late in life, is enough to guide the development of both effects.

## Methods

### Participants

In each experiment (Bouba-Kiki and SWI) we tested three different groups: Cataract-treated individuals, early blind individuals, and sighted controls. In the Bouba-Kiki experiment, we tested 23 cataract-treated patients (10 females/13males, mean age: 11.2y, range: 5–18y, mean age at surgery: 9.8y, range: 2–16y, mean time since surgery: 1.4y, range = 1d to 10y, mean visual acuity after surgery (measured as contrast sensitivity function (CSF) cutoff frequency): 4.9 cycle per degree, cpd). Prior to surgery all participants had been diagnosed with dense, bilateral, congenital cataracts and none had any pattern vision. For details on their visual abilities prior to surgery see Supplementary Table S1 in the supplementary information. Moreover, a group of early blind individuals (n = 17, 8 females/9 males, mean age: 11.2y, range: 7–16y), and a group of typically sighted controls (n = 124, thereof n = 71, 36 female/35 male, mean age = 11.9y, range = 4–16y in the visual condition and n = 53, 18 female/35 male, mean age = 10.5y, range = 4–17y in the haptic condition, see Procedure) took part. All cataract-treated patients and blind participants were tested in Ethiopia. Controls were either tested in Ethiopia (n = 26) or in Germany (n = 98).

In the SWI experiment, we tested 26 cataract-treated patients (13 female/13 male, mean age: 11.9y, range: 6-18y, mean age at surgery: 11.1y, range: 4–16y, visual acuity: 3.5 cpd; cf. Supplementary Table S1 for details). Additionally, a group of early blind individuals (n = 17, 8 females/9 males, mean age: 11.2y, range: 7–16y), and a group of typically sighted controls (n = 110, thereof n = 54 in the visuo-haptic condition, 20 female/34 males, mean age = 12.9y, range = 6–30y; and n = 56 in the haptic condition, 35 females/21 male, mean age = 13.5y, range = 6–29y) took part in the experiment. All cataract-treated patients and blind participants were tested in Ethiopia. Controls were either tested in Ethiopia (n = 6) or Germany (n = 104). To rule out that any differences found were caused by language, culture, or language barriers, results from Ethiopian and German controls were compared. As expected, performance of controls from the two countries did not differ in any of the two experiments. Thus, control groups from both countries were combined for the analyses in each experiment.

Fourteen of the cataract-treated patients and all blind participants took part in both experiments. All cataract-treated patients were previously diagnosed with mature, feremature, or partially absorbed bilateral cataracts. These were classified congenital (i.e. present at birth or shortly afterwards), as the patients showed nystagmus, a sign of visual deprivation in early life. Additionally, high familial incidences in some cases pointed to hereditary cataracts and most parents reported that their children had “white eyes” since birth. The patients were ophthalmologically examined and cataracts were surgically removed and treated with an intraocular lens implantation at Referral Hospital in Hawassa, Ethiopia. The target refraction was adjusted for far vision. All surgeries took place years after birth.

Ethiopian participants were tested either in the Hawassa Referral Hospital, in the Shashamane Catholic School for the Blind, or in the Sebeta Blind School in Ethiopia. German participants were tested in German primary and secondary schools or at Ulm University.

The study was performed in accordance with the Declaration of Helsinki and the ethics committee of Bielefeld University (EUB 2015-139) approved the study. Informed consent was obtained from participants, or from participant’s parents, or legal guardians in case of minors. All participants were naïve to the objective of the study.

### Experimental procedure

We investigated the sound-shape correspondence in the visual modality in cataract-treated participants and in sighted controls, and in the haptic modality in blind participants and sighted controls. In the visual condition of the Bouba-Kiki experiment a rounded and a spiky shape (Fig. 1A) were presented visually to the participant by the experimenter. The shapes were presented simultaneously, one to the left and one to the right side of the participant’s midline in a distance between 25 and 40 cm. The shapes were 3D-printed and consisted of black plastic material. Their size was 7.5 × 7 cm × 4.3 mm. To improve the contrast and make the different shapes as visible as possible for the cataract-treated patients, the shapes were presented on a white background. Participants were not allowed to touch the shapes. The experimenter told participants they would have to name the two shapes, knowing that one was named Bouba and the other Kiki. After asking the participant to repeat the two names, one after the other, the experimenter asked them to indicate which one they believed was named Bouba and which one Kiki. The left–right position in which the shapes were presented was counterbalanced across participants.

In the haptic condition of the experiment, the participants were blindfolded. The same shapes as in the visual condition were used. The shapes were presented to participants one after the other (in counterbalanced order across participants). Participants were allowed to explore each shape with two hands for a few seconds. After exploration, the experimenter placed each of the two shapes in one of the participant’s hands, simultaneously (with left–right shape position counterbalanced across participants) and asked them to report which one they believed was named Bouba and which Kiki (as described above). For the statistical analyses, the consensual association (i.e. “Bouba” as the round shape, and “Kiki” as the spiky shape) was assigned one point and the opposite choice was assigned zero points. Each participant took part in the experiment only once and thus only participated in either the visual or the haptic task.

For the SWI experiment, a large (60 × 60 × 55 mm) and a small (60 × 60 × 25 mm) cube made from light colored softwood was used. To obtain an identical weight (112 g), the cubes were weighted with metal inlays of different thickness, invisible from the outside. The centrally attached inlays were of circular shape so that the weight was evenly distributed around the center of mass within each object. To test for the visuo-haptic size-weight illusion, participants were asked to lift their hands off the table and turn their palms upwards, so that they could hold the objects unsupported. Then, each object was simultaneously placed in one of the hands of the participants (counterbalanced across participants). Participants were requested to enclose the objects with their hands. Then they were asked which object felt heavier in half of the cases, and which one felt lighter in the other half, counterbalanced across participants. The experimental procedure in the haptic task was identical, except that participants were blindfolded when entering the room, before seeing the objects. For the statistical analyses, the consensual allocation (i.e. the large object felt lighter than the small object) was assigned one point and the opposite choice was assigned zero points. Each participant took part in the experiment exactly once and thus only participated in either the visuo-haptic or the haptic task.

### Statistical analyses

All statistical analyses were performed with R version 4.1.2^57^ using the R packages plyr (v1.8.6) and ggplot2 (v3.3.5). The alpha level was set to 0.05. All tests were performed two-sided. We analyzed the data with logistic regression models. We assessed whether the probability of consensuall choices differed between groups separately for both experiments and conditions, respectively. Additionally, we analyzed whether within each group the probability of a consensual choice was different from chance level (i.e. 0.5). We also analyzed whether the choice was predicted by age (as a continuous variable) and, in the haptic condition of the size-weight illusion task, by age group (younger vs older than 12 years of age).

## Supplementary Information

Supplementary Information.

## Data Availability

The datasets generated during and/or analysed during the current study are available from the corresponding author on reasonable request.
