# Supplementary material for: Visual experience shapes the Bouba-Kiki effect and the size-weight illusion upon sight restoration from congenital blindness
Source: Sci Rep. 2023 Jul 15;13:11435. doi: 10.1038/s41598-023-38486-y (PMC10349879; doi:10.1038/s41598-023-38486-y)
Supplement: Supplementary file 1 — Supplementary Information. [file 41598_2023_38486_MOESM1_ESM.pdf]

Visual experience shapes the Bouba-Kiki effect and the Size-weight illusion upon sight restoration from congenital blindness

Sophia Piller*[1,2], Irene Senna*[1,3], Marc O. Ernst[1]

*These authors contributed equally to this work.

[1]Applied Cognitive Psychology, Faculty for Computer Science, Engineering, and Psychology, Ulm University, 89081 Ulm, Germany

[2]Transfer Center for Neuroscience and Education (ZNL), Ulm University, Parkstraße 11, 89073 Ulm, Germany

[3]Department of Psychology, Liverpool Hope University, Liverpool, United Kingdom

Corresponding author: Sophia Piller, sophia.piller@uni-ulm.de

Supplementary Table

Patient	Sex	Experiment	Age (years) BK /SWI	Pre-op CSF cutoff (cpd)	Post-op CSF cut-off (cpd) BK/ SWI	Time since surgery (years) BK/ SWI
p44	m	BK	5.0	unknown	1.84	1.08
p41	m	BK	6.0	unknown	12.43	1.08
p76	m	SWI	6.5	0.36	2.34	0.45
p54	f	BK	8.0	2.63	3.26	0.58
P74	f	SWI	8.0	0.04	0.04	0.01
p61	f	BK/ SWI	8.2/ 8.2	1.31	6/ 6	0.01/ 0.01
p35	f	SWI	8.8	unknown	2.1	4.13
p77	f	SWI	9.0	0.45	1.86	0.01
p62	f	BK/SWI	9.0 / 9.0	0.04	0.09/ 0.09	0.01 / 0.01
p67	m	BK /SWI	9.0/ 9.0	1.51	1.41 /1.41	0.01/ 0.01
p47	m	BK /SWI	9.1/ 9.4	0.71	3.02/ 5.37	0.16/ 0.52
p12	f	BK	10.0	unknown	3.68	3.11
P69	f	SWI	10.0	0.04	0.08	0.01
p63	m	SWI	10.2	5.21	3.16	0.01
p56	f	BK / SWI	10.1/ 10.4	2.66	4.05/ 5.3	0.16/ 0.52
p60	f	BK/ SWI	10.0/ 10.4	2.84	5.82/ 9.54	0.01/ 0.36
P50	m	SWI	10.6	3.77	5.31	1.61
p24	m	BK	11.0	unknown	2.38	2.64
p11	m	BK	11.0	unknown	7.76	3.11
p01	f	BK	11.0	unknown	3.6	4.03
p75	f	SWI	11.0	0.23	0.21	0.01
p57	m	BK	11.1/ 11.4	1.89	2.3/ 1.43	0.16/ 0.52
P53	m	SWI	11.6	0.72	1.49	1.61
p58	m	BK	12.0	unknown	13.27	10.00
p33	m	BK	13.0	2.2	12.05	1.82
p72	m	SWI	13.2	0.39	5.68	0.01
p48	f	BK/ SWI	13.4/ 13.8	0.88	2.72/ 4.64	0.16/ 0.52
p37	m	BK/ SWI	13.0/ 15.1	3.69	5.60/ 6.59	1.08/3.11
p65	m	BK/SWI	14.2/ 14.2	6.33	6.03/ 6.03	0.01/ 0.01
p66	m	BK/ SWI	15.2/ 15.2	1.5	1.58/ 1.58	0.0/ 0.0
p59	f	BK/ SWI	15.2/ 15.2	2.91	4.72/4.72	0.0/ 0.0
p68	m	BK/ SWI	15.2/ 15.2	0.08	1.3/ 1.3	0.0/ 0.0
P30	f	SWI	15.8	0.25	1.59	2.83
P39	m	SWI	18.1	2.64	2	2.10
p29	f	BK/ SWI	18.8/ 18.8	0.27	11.99/ 11.99	2.83/ 2.83

Supplementary Table S1. Clinical characteristics of the cataract-treated patients.

The table displays sex (*f* indicates female, *m* indicates male), type of experiment (*BK* indicates Bouba-Kiki, *SWI* indicates size-weight illusion), age at test (in years), visual acuity prior and after surgery (in cycles per degree, cpd, measured as contrast sensitivity function (CSF) cutoff frequency as described in [36]), and time that has passed since surgery (in years) of the tested cataract-treated patients.
